# *Btg1* is Required to Maintain the Pool of Stem and Progenitor Cells of the Dentate Gyrus and Subventricular Zone

**DOI:** 10.3389/fnins.2012.00124

**Published:** 2012-08-30

**Authors:** Stefano Farioli-Vecchioli, Laura Micheli, Daniele Saraulli, Manuela Ceccarelli, Sara Cannas, Raffaella Scardigli, Luca Leonardi, Irene Cinà, Marco Costanzi, Maria Teresa Ciotti, Pedro Moreira, Jean-Pierre Rouault, Vincenzo Cestari, Felice Tirone

**Affiliations:** ^1^Institute of Cell Biology and Neurobiology, National Research CouncilFondazione Santa Lucia, Rome, Italy; ^2^Department of Human Sciences, LUMSA UniversityRome, Italy; ^3^Institute of Translational Pharmacology, National Research Council, Fondazione EBRI Rita Levi-MontalciniRome, Italy; ^4^Mouse Biology Unit, European Molecular Biology LaboratoryMonterotondo, Italy; ^5^Institut de Génomique Fonctionnelle de Lyon, Ecole Normale Supérieure de Lyon, Université Lyon1, CNRS UMR 5242, INRA UMR1288Lyon, France

**Keywords:** BTG family, differentiation, knock out mice, learning and memory, neural stem cells, neurogenic niches, proliferation

## Abstract

Btg1 belongs to a family of cell cycle inhibitory genes. We observed that *Btg1* is highly expressed in adult neurogenic niches, i.e., the dentate gyrus and subventricular zone (SVZ). Thus, we generated *Btg1* knockout mice to analyze the role of *Btg1* in the process of generation of adult new neurons. Ablation of *Btg1* causes a transient increase of the proliferating dentate gyrus stem and progenitor cells at post-natal day 7; however, at 2 months of age the number of these proliferating cells, as well as of mature neurons, greatly decreases compared to wild-type controls. Remarkably, adult dentate gyrus stem and progenitor cells of *Btg1*-null mice exit the cell cycle after completing the S phase, express p53 and p21 at high levels and undergo apoptosis within 5 days. In the SVZ of adult (two-month-old) *Btg1*-null mice we observed an equivalent decrease, associated to apoptosis, of stem cells, neuroblasts, and neurons; furthermore, neurospheres derived from SVZ stem cells showed an age-dependent decrease of the self-renewal and expansion capacity. We conclude that ablation of *Btg1* reduces the pool of dividing adult stem and progenitor cells in the dentate gyrus and SVZ by decreasing their proliferative capacity and inducing apoptosis, probably reflecting impairment of the control of the cell cycle transition from G1 to S phase. As a result, the ability of *Btg1*-null mice to discriminate among overlapping contextual memories was affected. *Btg1* appears, therefore, to be required for maintaining adult stem and progenitor cells quiescence and self-renewal.

## Introduction

During neurogenesis post-mitotic neurons of distinct types are generated from neural stem/progenitor cell (NSPs), both in the developing brain and in the adult brain niches of the hippocampus dentate gyrus and subventricular zone (SVZ), through progressive steps of cell cycle exit, differentiation, and migration (Gage, 2000; Ming and Song, 2005; Kriegstein and Alvarez-Buylla, 2009). Cell cycle arrest and neurogenesis are highly coordinated and interactive processes, governed by cell cycle genes and neural transcription factors. The expression of proneural genes, which convert undifferentiated precursors into neurons, is also linked to a negative control of the cell cycle (Farah et al., 2000). One molecule coordinating cell cycle exit with differentiation in neural progenitor cells is the transcriptional coregulator PC3/*Tis21* (also referred to as *Btg2*), whose ablation enhances the proliferation of adult hippocampal granule progenitor cells and also impairs their terminal differentiation (Farioli-Vecchioli et al., 2009). Conversely, overexpression of *PC3*/*Tis21* in hippocampal progenitor cells accelerates their differentiation (Farioli-Vecchioli et al., 2008).

Adult hippocampal neurogenesis occurs in the subgranular zone of the dentate gyrus from putative neural stem cells with radial glial-like morphology, identified by the expression of GFAP in their processes or also of nestin and Sox2, and defined as type-1 cells (Seri et al., 2001; Filippov et al., 2003; Fukuda et al., 2003; Graham et al., 2003; Kempermann et al., 2004; Komitova and Eriksson, 2004). Type-1 cells evolve into proliferating progenitor cells, namely type-2, which exist in two subtypes 2a and 2b, both nestin-positive, one negative and one positive for the immature neuronal marker doublecortin (DCX; Fukuda et al., 2003; Kronenberg et al., 2003) and type-3, which then become post-mitotic granule neurons, attaining stage 5 and 6 (Kempermann et al., 2004). This stage is denoted by the expression of the mature, post-mitotic neuronal marker NeuN, which coexists initially with DCX (Kempermann et al., 2004). On the other hand, SVZ, the other adult neurogenic brain region (Alvarez-Buylla and Lim, 2004), comprises type B stem astrocytic-like cells, type C transit amplifying cells and type A migrating neuroblasts (Lagace et al., 2007; Zhao et al., 2008).

The hippocampus is known to be required in the formation of spatial and associative memories, a process in which a specific role appears to be played by the new neurons continuously generated during adulthood from progenitor cells (Frankland and Bontempi, 2005; Bird and Burgess, 2008; Deng et al., 2010). In fact, impairment of differentiation of hippocampal progenitor cells in *PC3*/*Tis21*-null mice profoundly affects their function in hippocampus-dependent contextual memory circuits and tasks (Farioli-Vecchioli et al., 2008, 2009). Notwithstanding the pan-neural expression of *PC3*/*Tis21* and these profound cognitive effects following its ablation, no lethal phenotype is observed, suggesting that other related genes may produce a redundant control of differentiation.

In this regard, B-cell translocation 1 gene (*Btg1*) belongs to the gene family comprising *PC3*/*Tis21*, *BTG3*, *TOB*, and *TOB2*. It was originally identified as a sequence associated to a chromosomal translocation in a lymphoid malignancy (Rouault et al., 1992). *Btg1* shares with *PC3*/*Tis21* 65% protein identity and the antiproliferative properties (Rouault et al., 1992; Tirone, 2001). Moreover, *Btg1* induces avian myoblast differentiation (Marchal et al., 1995; Rodier et al., 1999) and the development of endothelial cells (Iwai et al., 2004) and is also likely involved in the differentiation of sperm cells (Raburn et al., 1995). *Btg1* is expressed in the developing and adult brain (Su et al., 2004; Kamaid and Giráldez, 2008), but no information on its function in neural tissues is available. Thus, we generated *Btg1* knock out mice and analyzed the functional contribution by *Btg1* to the adult neurogenic niches of the hippocampus and SVZ (Zhao et al., 2008). It turned out that *Btg1* is necessary for the maintenance and generation of progenitor cells and new neurons of both regions, because its ablation was associated with a massive apoptosis of stem and progenitor cells, probably a result of the loss in the control by *Btg1* of the cell cycle progression from G1 to S phase. Consequently, the number of new dentate gyrus neurons generated was largely reduced in mice lacking *Btg1*. This decrease of new neurons had a selective effect on hippocampus-dependent memory, as it specifically affected the ability to discriminate between similar contexts (pattern separation).

## Materials and Methods

### Construction of m*Btg1* targeting vector

A mouse genomic clone was isolated from 129/Sv mouse library of phage lambda by standard techniques. A fragment of 6 kB encompassing the mouse *Btg1* gene was cloned in pBluescript II. A phosphoglycerate kinase-neomycin resistance cassette was inserted in the *Sac*II restriction site located in mouse *Btg1* exon 1 (49 bp after ATG). A Herpes simplex virus thymidine kinase gene cassette (negative selection) was cloned adjacent to the 3′ end of the genomic region.

### Generation of targeted ES cells and of *Btg1*-null mice; genotyping

We proceeded as described previously (Berthet et al., 2004), by electroporating embryonic stem cells (ES) with the linearized targeting vector and selecting them with G418 (250 μg/ml) and ganciclovir (0.5 μg/ml). A resistant ES cells clone was injected into 3.5-day C57BL/6 blastocysts to obtain male chimeras.

The genotype of resistant ES cells and of agouti pups was determined, following digestion of DNA with *Acc*I, by Southern blotting using as probes a genomic fragment of about 0.5 kb comprising the *Eco*RI-*Bgl*II region at 5′ of the gene or a fragment of the neomycin sequence (wild-type or knockout alleles generated 5 or 6.1 kb fragments, respectively). Genotyping of mice was routinely performed by PCR, using genomic DNA from tail tips. Three primers were used to identify mice carrying the different genotypes *Btg1*^−/−^, *Btg1*^+/−^ or *Btg1*^+/+^, one complementary to the neo cassette (m*Btg1*-Neo-R 5′-CGGAGAACCTGCGTGCAATC-3′) and the other two complementary to the targeted exon I (m*Btg1*-F 5′-CCATGCATCCCTTCTACACCC-3′; m*Btg1*-R 5′-TGCAGGCTCTGGCTGAAAGT-3′) and were amplified together in the PCR reaction to obtain patterns of amplification specific for each of the three combinations of alleles (knockout, 388 bp amplification by m*Btg1*-F and m*Btg1*-Neo-R primers; wild-type, 136 bp amplification of exon I by m*Btg1*-F and m*Btg1*-R primers). Mice were maintained under standard specific-pathogen-free conditions, and underwent behavioral testing during the second half of the light period (between 2:00 and 5:00 p.m.) in sound insulated rooms.

All animal procedures were completed in accordance with the Istituto Superiore di Sanita’ (Italian Ministry of Health) and current European (directive 2010/63/EU) Ethical Committee guidelines. Btg1 knockout mice are available upon request to J.-P. Rouault.

### BrdU treatment of mice and sample preparation for immunohistochemistry

In post-natal day 60 (P60) *Btg1*^+/+^ and *Btg1*^−/−^ mice, 1- to 5-day-old neurons in the dentate gyrus and SVZ were detected by bromodeoxyuridine (BrdU) incorporation, after treatment with five daily injections of BrdU (95 mg/kg i.p.), from P55 to P59, followed by perfusion at P60 (Farioli-Vecchioli et al., 2008; see Figures 3C, 6B, and 7B). Similarly, 28-day-old neurons in the dentate gyrus and olfactory bulb were detected in *Btg1*^+/+^ and *Btg1*^−/−^ mice after treatment with five daily injections of BrdU (95 mg/kg i.p.) from P55 to P59, followed by perfusion at P83 (see Figures 3D and 7C); 28-day-old neurons in the olfactory bulb were also analyzed at an earlier age: treatment with BrdU from P5 to P9, followed by perfusion at P33 (Figure 7C). To detect progenitor cells in the dentate gyrus entering the S phase, P60 or P7 mice were perfused 2 h after treatment with BrdU (a single injection), according to previous protocols (Arguello et al., 2008; Figures 4B,C). To detect progenitor cells in the dentate gyrus that have entered the S phase within the 20- or 48-h preceding analysis, P60 mice underwent a single injection of BrdU 20 or 48 h before perfusion (Figure 6D). Brains were collected after transcardiac perfusion with 4% paraformaldehyde (PFA) in PBS – DEPC and kept overnight in PFA. Afterward, brains were equilibrated in sucrose 30% and cryopreserved at −80°C.

### Immunohistochemistry

Immunohistochemistry was performed on serial freefloating sections cut at 40 μm thickness for hippocampus as well as for the SVZ and olfactory bulb, at −25°C in a cryostat from brains embedded in Tissue-Tek OCT (Sakura, Torrence, CA, USA). Sections were then stained for multiple labeling using fluorescent methods. BrdU incorporation was detected following pretreatment of sections to denature the DNA, with 2N HCl 45 min at 37°C and then with 0.1 M sodium borate buffer pH 8.5 for 10 min.

Primary antibodies used were a rat monoclonal antibody against BrdU (AbD Serotech, Raleigh, NC, USA; MCA2060; 1:400), mouse monoclonal antibodies raised against Nestin (Chemicon International; MAB353; 1:50), NeuN (Chemicon International; MAB377; 1:300), PH3 (Cell Signaling Technology, Danvers, MA, USA; 9706; 1:100), or against p53 (Abcam, Cambridge, UK; ab26; 1:100), a rabbit monoclonal antibody against Ki67 (LabVision Corporation, Fremont, CA, USA; SP6; 1:200), rabbit polyclonal antibodies against cleaved (activated) Caspase-3 (Cell Signaling Technology, Danvers, MA, USA; 9661; 1:100), p21 (Santa Cruz Biotechnology, Santa Cruz, CA, USA; Sc-397, 1:100), or against goat polyclonal antibodies raised against GFAP (Santa Cruz Biotechnology; Sc-6170, 1:300) or DCX (Santa Cruz Biotechnology; Sc-8066, 1:300). Secondary antibodies used to visualize the antigen were either a donkey anti-rat monoclonal antiserum conjugated to Cy2 or TRITC (tetramethylrhodamine isothiocyanate; Jackson ImmunoResearch, West Grove, PA, USA; BrdU), or a donkey anti-mouse antiserum conjugated to Cy2, TRITC, or Alexa 647 (Invitrogen, San Diego, CA, USA; Nestin, NeuN, PH3, p53), or a donkey anti-rabbit antiserum conjugated to TRITC or to Cy2 (Jackson ImmunoResearch; Ki67, Caspase-3, p21), or a donkey anti-goat antiserum conjugated to Cy2, TRITC, or Alexa 647 (Invitrogen, San Diego, CA, USA; GFAP, DCX, p21).

Images of the immunostained sections were obtained by laser scanning confocal microscopy using a TCS SP5 microscope (Leica Microsystem). Analyses were performed in sequential scanning mode to rule out cross-bleeding between channels.

### Quantification of cell numbers and volumes

Stereological analysis of the number of cells was performed by analyzing with confocal microscopy one-in-six series of 40-μm freefloating coronal sections (240 μm apart), to count cells expressing the indicated marker throughout the whole rostrocaudal extent of the dentate gyrus. The total estimated number of cells within the dentate gyrus, positive for each of the indicated markers, was obtained multiplying the average number of positive cells per section by the total number of 40-μm sections comprising the entire dentate gyrus (about 50–60 sections), as described (Gould et al., 1999; Jessberger et al., 2005; Kee et al., 2007; Farioli-Vecchioli et al., 2008). Three animals per group were analyzed. Cell numbers in the SVZ and in the olfactory bulb were obtained similarly, by counting cells visualized with confocal microscopy throughout the whole rostrocaudal extent of these structures in one-in-six series of 40-μm freefloating coronal sections (240 μm apart). Cell number obtained for each SVZ and olfactory bulb section was divided for the corresponding area of the section, as described (Colak et al., 2008), in order to obtain the average number of SVZ or olfactory bulb cells per square millimeter. Areas were obtained by tracing the outline of the whole SVZ, or olfactory bulb, identified by the presence of cell nuclei stained by Hoechst 33258 on a digital picture captured and measured using the I.A.S. software (Delta Sistemi, Rome, Italy). Three animals per group were analyzed. The I.A.S. software was also used to count labeled cells. The volume of the dentate gyrus and hippocampus was calculated multiplying the average dentate gyrus area by section thickness and by number of sections (one-in-six series of 40-μm coronal sections).

### *In situ* hybridization

Preparation of sections and hybridization were performed as reported previously (Canzoniere et al., 2004). An antisense riboprobe detecting *Btg1* mRNA was synthesized by SP6 polymerase from the pcDNA3-m*Btg1* vector, in whose *Hin*dIII5′-*Eco*RI3′ sites we cloned the 3′ UTR region of mouse *Btg1* mRNA (nt 1210–1730). The cloned *Btg1* 540 bp long sequence, which is part of the second exon of *Btg1* and is devoid of cross-homologies, was amplified using genomic mouse DNA as template and was checked by sequencing. Riboprobes were labeled with digoxigenin-UTP (Transcription kit; Roche Products), following the protocol of the manufacturer. No signal was detected by the sense probe.

### Detection of senescent progenitor cells and neurons by β-Galactosidase staining

Senescent progenitor cells were identified by detecting β-Galactosidase activity at pH 6 as described (Dimri et al., 1995) by means of Senescence staining kit (Cell Signaling Technology, Danvers, MA, USA), following the protocol of the manufacturer.

### Neural stem cell cultures

Neural stem cells cultures were performed as described by Gritti et al. (2001). Two-month-old mice (wild-type or *Btg1*-null) were euthanized by cervical dislocation and the brains were removed. SVZ were dissected out and cells were isolated by enzymatic digestion (1.33 mg/ml trypsin, 0.7 mg/ml hyaluronidase, and 0.2 mg/ml kynurenic acid) for 30 min at 37°C and mechanical dissociation with small-bore Pasteur pipette. Neurospheres were grown in a humidified incubator at 37°C in 5% CO_2_ and cultured in DMEM/F12 medium supplemented with B27 and EGF and bFGF (20 and 10 ng/ml, respectively). Cells were passaged every 4th day by mechanically dissociating neurospheres into single cells.

#### Neurosphere assay

Cells isolated from SVZ were cultured under clonal conditions, in which it has been reported that neurospheres are generated from single cells and serve as an index of the number of *in vivo* neural stem cells (Morshead et al., 2003; Kippin et al., 2005). Cells were plated at 10 cells/μl in 24-well (0.5 ml/well) uncoated plates in growth medium. The total number of neurospheres was counted after 7 days *in vitro* (7 DIV).

#### Expansion capacity

Primary neurospheres were dissociated into single cells and plated at the same clonal density. Then, secondary neurospheres were dissociated and the number of cells was determined and expressed as average expansion from the initial starting population (number of cells from secondary neurospheres at 7 DIV/number of seeded cells). The size of neurospheres was expressed as a volume calculated after measuring their diameter in phase contrast pictures (assuming a spherical shape).

For the growth curve, 8000 cells from wild-type and knockout neurospheres at passage 5 were seeded in 24-well plates. At each subculture passage (every 7 days) the viable cells were counted and totally re-plated under the same conditions.

#### Pair cell assay

The Pair cell assay was performed as described by Bultje et al. (2009). Single cells isolated from SVZ of P7 or 2-month-old mice (wild-type and *Btg1*-null) were plated at clonal density on poly-d-lysine (Sigma Aldrich; St. Louis, MO, USA) coated coverslips in 24-well plates. After 24 h the cells were fixed in 4% paraformaldehyde and immunostained with goat polyclonal anti-GFAP (Santa Cruz Biotechnology; Sc-6170, 1:200) and mouse monoclonal anti-TuJ1 (Covance, USA; 1:250) antibodies. Secondary antibodies used were donkey anti-mouse TRITC and donkey anti-goat Cy2 (Jackson ImmunoResearch). Nuclei were counterstained with Hoechst. Coverslips were mounted on slides and imaged at confocal microscopy (Leica, TSP). The number of progenitor pairs was determined by counting at least 60 pair cells per mice (at least three mice per genotype).

#### Immunofluorescence on neurospheres and microscopy

For active caspase-3 (Ser-15) immunostaining, neurospheres were plated on matrigel-coated coverslips and then fixed in 4% paraformaldehyde for 10 min at RT. After fixation, neurospheres were permeabilized in 0.1% Triton X-100 in PBS and then incubated with the antibody against active caspase-3 (Cell Signaling Technology).

Immunostained neurospheres were mounted in Aquapolymount and analyzed at confocal microscopy (Leica, TSP). Z-stacks images were captured at 1 mm intervals with a 40× objective and a pinhole of 1.0 Airy unit. The numbers of caspase positive cells were counted as a percentage of Hoechst positive-nuclei in four non-adjacent Z-stacks images per neurosphere.

### Reverse transcription-PCR; genomic DNA southern analysis

Total mRNA from neurospheres was extracted and analyzed by semiquantitative reverse transcription (RT-PCR) as described previously, with minor modifications (Canzoniere et al., 2004). Briefly, 10 μg of total RNA were treated with DNase (RQ1; Promega, Madison, WI, USA), denatured at 75°C for 5 min, and added to a final reaction volume of 50 μl. Half of the reaction volume was then incubated for 2 h at 37°C with Moloney murine leukemia virus-RT (Promega). The remaining half of the volume without RT was used as negative control in PCR amplifications for possible contamination by genomic DNA. Two microliters of each RT reaction were then used for PCR amplification, using primers amplifying the region between the end of the first exon and beginning of the second exon of *Btg1*. The 18S RNA was coamplified to measure the efficiency of the reaction and the RNA amount in each sample. The amplified products were visualized by agarose gel electrophoresis.

To define the structure of the recombined *Btg1* locus, Southern blot analysis was performed using genomic DNA extracted from tail tips. Twenty micrograms of genomic DNA was restricted with *Acc*I, gel separated, blotted to a nylon filter, and hybridized with a [^32^P]-labeled probe, whose sequence encompasses the 5′ region of the *Btg1* gene external to the targeting vector.

### Behavioral tests

*Btg1*-null (*n* = 30) and wild-type (*n* = 30) male mice aged between 2 and 4 months were used for behavioral evaluation. All of them were preliminarily tested in an open field, to assess locomotion and anxiety-related behaviors. Mouse activity was recorded for 15 min and the distance traveled, moving speed, rearing events and relative occupancy of external vs. central sectors of the arena were analyzed. No significant differences between genotypes were observed in any of these variables (data not shown). Additionally, a plus maze test was performed, in which the animals were allowed to explore the apparatus (a cross-shaped maze placed 60 cm from the ground, with four arms 30 cm long) for 5 min and the relative occupancy of closed vs. open arms was evaluated. Again, no significant differences between genotypes were observed (data not shown).

#### Water maze

A delayed-matching-to-place version of the task was conducted as previously described (Chen et al., 2000), with minor modifications. In a pool measuring 130 cm in diameter, mice were trained to navigate to a hidden platform (10 cm in diameter) until reaching a rigorous performance criterion of three consecutive trials with an average escape latency of less than 20 s, or completing a maximum of 24 trials. This was repeated until a total of four different platform locations were learned. The behavior of mice was analyzed by EthoVision software (Noldus Information Technology, Wageningen, NL, USA).

#### One-trial contextual fear conditioning

Conditioning was performed in a training chamber (A) with a single footshock (2 s; 0.7 mA) delivered 180 s after placement of a mouse into the chamber. Mice were left in the chamber for a further period of 20 s and then returned to their home cage. Contextual test (5 min) was performed 24 h after training, in the same chamber. Forty-eight hours after training, mice were tested in a different context (C). The amount of freezing was assessed off-line by an experimenter blind to the genotypes of the animals.

### Contextual fear-discrimination learning

The test was conducted as previously described (Sahay et al., 2011a), with minor modifications. Conditioning was performed in the training chamber (A). Starting from the next day, mice were exposed, on a daily basis, to both the training context, in which they continued to receive a single footshock, and a similar context (B), in which they were never shocked. Freezing was measured each day in both the contexts, to evaluate continuously the discrimination level.

## Results

### In adult mouse brain *Btg1* is expressed in the dentate gyrus, SVZ, olfactory bulb, and cerebellum

**Figure 1:**
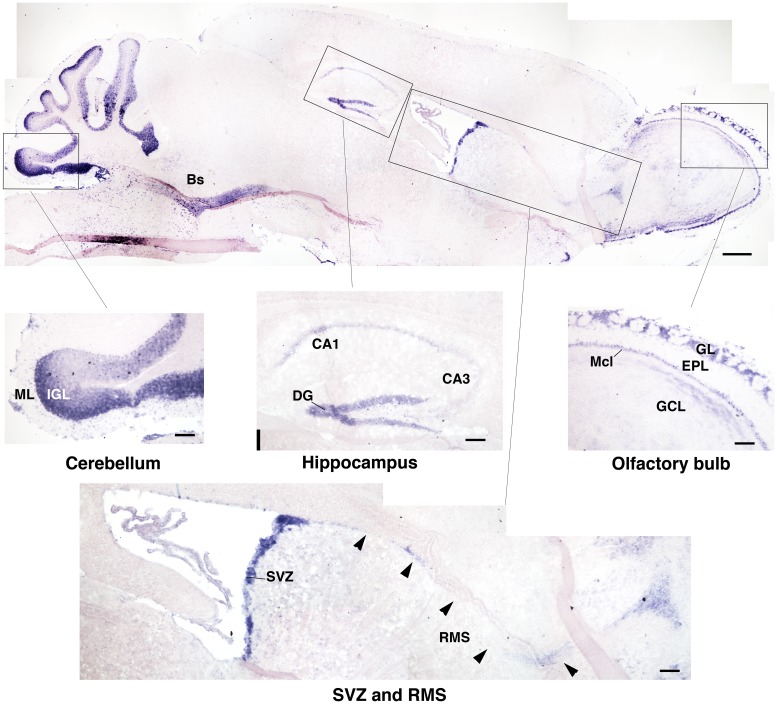
**Expression of Btg1 in the mouse adult brain**. A representative sagittal section of the brain from a 2-month-old mice, showing the expression of *Btg1* mRNA labeled by *in situ* hybridization. *Btg1* mRNA is clearly detectable (see enlargements of boxed areas): (i) in all neurons within the cell layers in the dentate gyrus blades of the hippocampus (DG) and to a lower extent in CA3 and CA1; (ii) in the subventricular zone (SVZ) and in neurons migrating from it along the rostral migratory stream (RMS); (iii) in the olfactory bulb in the glomerular layer (GL) and in the mitral cell layer (Mcl), while it is present to a lower level in the granule cell layer (GCL) and is absent in the external plexiform layer (EPL); (iv) in the cerebellum, in the molecular layer (ML) and the internal granular layer (IGL); (v) in the brainstem (Bs; upper panel). Scale bars: 500 μm (panel above) or 100 μm (enlargements).

**Figure 2:**
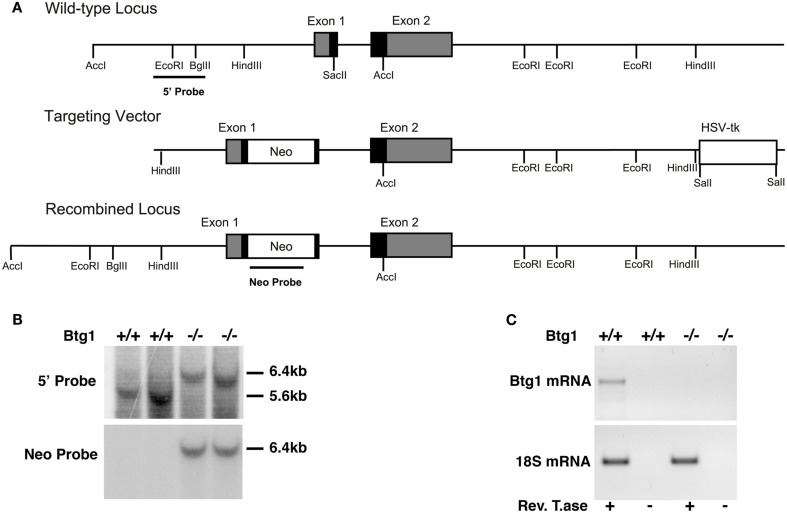
**Mouse Btg1 targeting in ES cells and generation of Btg1^−/−^ mice**. **(A)** Genomic organization and disruption strategy of the mouse *Btg1* gene; the gene, the targeting construct, and the recombined mouse *Btg1* allele are shown. Gray or black boxes: untranslated or translated regions, respectively. **(B)** Genomic Southern blot analysis of genomic DNA from wild-type and *Btg1*^−/−^ mice, digested with *Acc*I and hybridized to the 5′ and Neo probes. **(C)** Semiquantitative RT-PCR analysis of *Btg1* mRNA extracted from SVZ neurospheres of P60 mice with different *Btg1* genotypes. Equal amounts of RT-PCR products, amplified in the region encompassing part of the first and second *Btg1* exon or 18S mRNA were visualized by gel. RT± refers to the products of amplification performed in parallel on aliquots of each RNA sample, preincubated or not with ReverseTranscriptase, as negative controls.

As we were interested in investigating a possible role of *Btg1* in adult neurogenesis, we analyzed the expression of *Btg1* mRNA in the brain of 2-month-old mice by *in situ* hybridization. As shown in Figure 1, high levels of *Btg1* are detectable in the adult neurogenic niches, i.e., in the dentate gyrus of the hippocampus and in the SVZ, and also in the olfactory bulb, where neurons from the SVZ migrate along the rostral migratory stream (Zhao et al., 2008); moreover, a high expression was detectable in cerebellum, in the internal granular cell layer. The high expression of *Btg1* in adult brain neuroepithelia, i.e., where adult progenitor cells are generated, suggested that this gene may have a role in adult neurogenesis. To study this possibility we generated *Btg1*-null mice, with deletion of the first exon of the gene (Figure 2).

### In the absence of *Btg1*, adult neurogenesis in the dentate gyrus is impaired

Then, we sought to assess whether the generation of new neurons in the neurogenic adult niches of the hippocampus and SVZ is dependent on *Btg1* expression. We first analyzed the maturation of progenitor cells of the dentate gyrus in the adult hippocampus (P60) of *Btg1* knockout mice.

**Figure 3:**
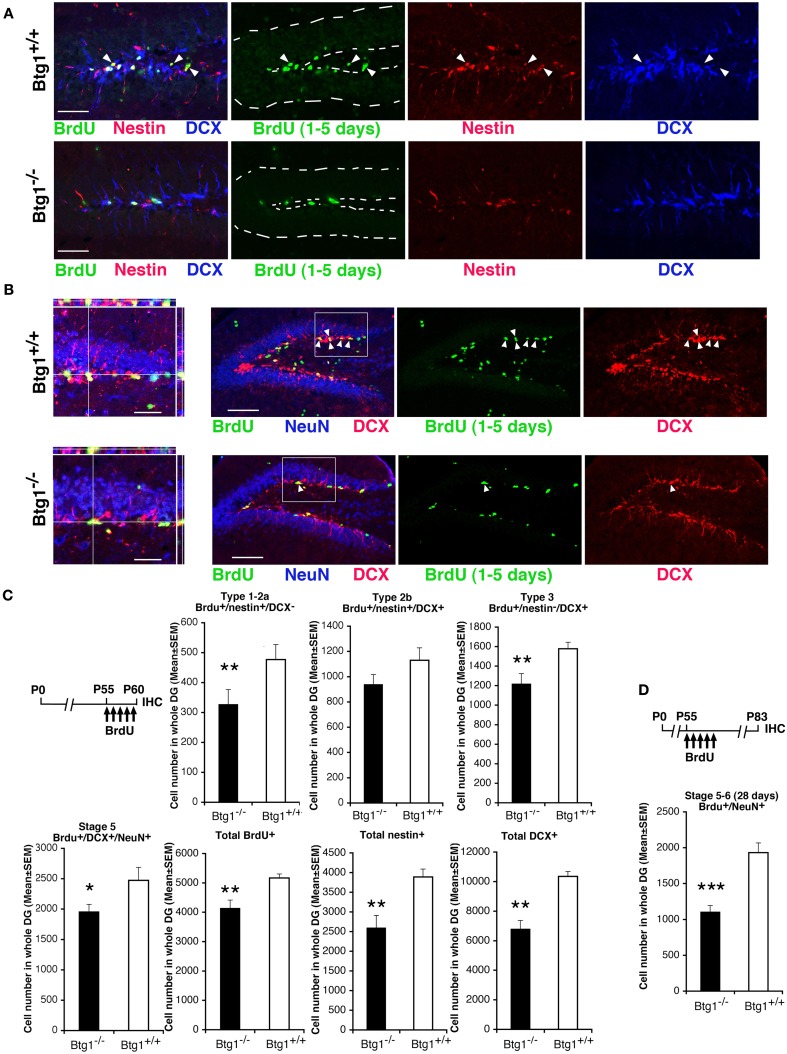
**In Btg1-null adult mice the number of new 1- to 5-day-old dentate gyrus progenitor cells and of 28-day-old neurons is reduced**. Representative images showing a decrease in the dentate gyrus of *Btg1*-null mice of **(A)** new stem and progenitor cells (type-1–2a; BrdU^+^/nestin^+^/DCX^−^, marked by green and red and negative to blue, respectively, indicated by white arrowheads; scale bar, 50 μm) and of **(B)** post-mitotic 1- to 5-day-old neurons (stage 5; BrdU^+^/DCX^+^/NeuN^+^, indicated within the box by white arrowheads; scale bar, 100 μm), as detected by incorporation of BrdU after five daily injections in P60 *Btg1*^+/+^ and *Btg1*^−/−^ mice, and by the specific markers indicated, through multiple-labeling confocal microscopy. **(B)** On the left: 3D reconstruction from Z-stack of triple- and double-positive cells shown in the boxed area (scale bar, 20 μm). In **(A)** the dentate gyrus is outlined by a broken line. **(C)** Scheme of BrdU treatment and quantification of the number of new 1- to 5-day-old type-1–2a (BrdU^+^/nestin^+^/DCX^−^), type-2b (BrdU^+^/nestin^+^/DCX^+^), and type-3 (BrdU^+^/nestin^−^/DCX^+^) stem and progenitor cell, as well as of stage 5 post-mitotic neurons, indicated a significant decrease (except for type-2b) in P60 *Btg1*-null mice. Also total BrdU-positive, total nestin-positive and total DCX-positive progenitor cells decreased significantly. **(D)** However, the highest reduction was observed for 28-day-old terminally differentiated neurons (BrdU^+^/NeuN^+^; above the graph is the scheme of treatment of mice with five BrdU injections 28 days before perfusion at P83). Cell numbers in dentate gyrus, shown in **(C,D)** were measured as described in Materials and Methods and are represented as mean ± SEM of the analysis of three animals per group. *p < 0.05, **p < 0.01, or ***p < 0.001 vs. *Btg1*^+/+^ dentate gyrus; Student’s *t*-test.

We identified new 1- to 5-day-old dentate gyrus progenitors and neurons by treating mice at P55 with five daily injections of BrdU, and analyzing them in the different cell populations of the dentate gyrus (Figures 3A–C). In *Btg1*-null mice we observed a significant decrease, with respect to control mice, in the number of 1- to 5-day-old type-1 stem and type-2a progenitor cells (BrdU^+^/nestin^+^/DCX^−^; *p* = 0.004; Figures 3A,C), while type-2b progenitor cells decreased but not significantly (BrdU^+^/nestin^+^/DCX^+^; Figures 3A,C). A significant decrease was evident also for 1- to 5-day-old type-3 progenitor cells (BrdU^+^/nestin^−^/DCX^+^; 22% decrease, *p* = 0.006; Figures 3A,C), that still express DCX but not nestin (Kronenberg et al., 2003), as well as for 1- to 5-day-old stage 5 terminally differentiated neurons expressing the late differentiation marker NeuN (BrdU^+^/DCX^+^/NeuN^+^; 20% decrease, *p* = 0.01; Figures 3B,C). In parallel, in *Btg1*-null mice a large and significant reduction of the whole population of 1- to 5-day-old new neurons occurred (total BrdU^+^; *p* = 0.001; Figure 3C), as well as of nestin^+^ (33% decrease, *p* = 0.001; Figures 3A,C) and DCX^+^ (34% decrease, *p* = 0.009; Figures 3A–C) stem/progenitor cells; the highest decrease, however, was observed for 28-day-old stage 5–6 terminally differentiated neurons (BrdU^+^/NeuN^+^, 42% decrease, *p* = 0.000; Figure 3D), indicating, as a whole, that a major decrease in the generation of new neurons occurred after ablation of *Btg1*. Furthermore, we tested whether the reduced number of new neurons was the result of non-specific changes, such as a reduced dentate gyrus volume. No significant difference was observed between *Btg1*^+/+^ and *Btg1*^−/−^ mice at P60 in the volumes of the dentate gyrus (0.27 ± 0.01 and 0.32 ± 0.03 mm^3^, respectively; *p* = 0.21, *n* = 3) or of the whole hippocampus (5.07 ± 0.19 and 5.60 ± 0.87 mm^3^, respectively; *p* = 0.52, *n* = 3).

Next we sought to ascertain whether the impairment of neurogenesis was dependent on a reduced ability of the pool of progenitor cells in the subgranular zone of the dentate gyrus to proliferate or to survive and generate new neurons.

### Ablation of *Btg1* reduces the pool of dividing adult progenitor cells in the dentate gyrus and induces their exit from the cell cycle within a few hours after completing the S phase

**Figure 4:**
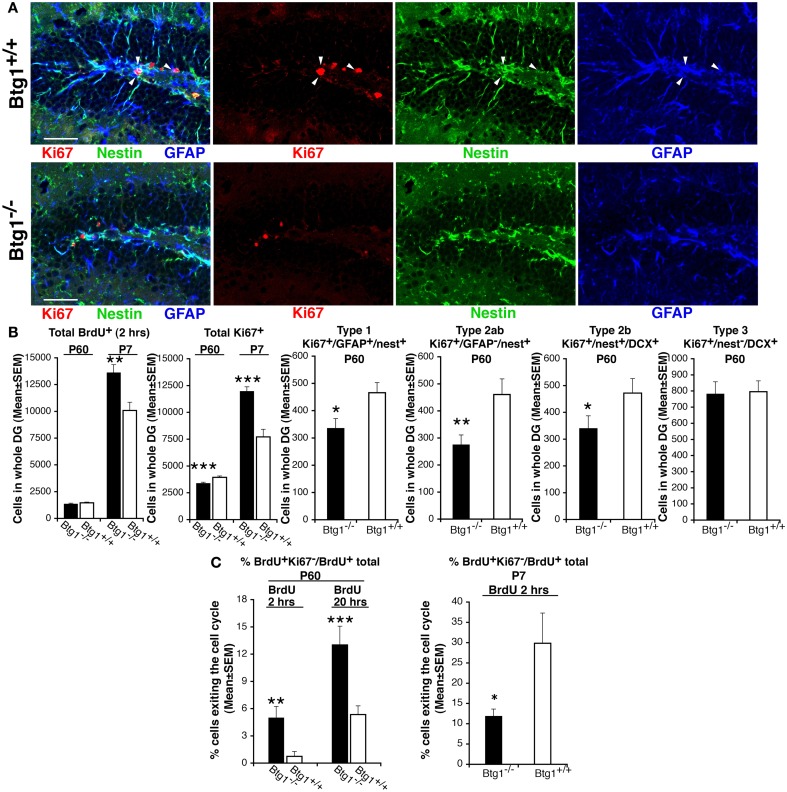
**In Btg1-null adult mice the number of cycling dentate gyrus progenitor cells decreases, after a transient early post-natal increase**. **(A)** Representative confocal images in P60 dentate gyrus *Btg1*-null mice showing a decrease of dividing stem cells, identified by means of Ki67 (type-1; Ki67^+^/nestin^+^/GFAP^+^, red, green, and blue, respectively, indicated by white arrowheads; scale bar, 50 μm). **(B)** The quantification of the total number of dentate gyrus cells entering the S phase (total BrdU^+^ cells after a 2-h pulse) did not show significant differences at P60, whereas at P7 their number increased significantly in *Btg1*-null mice. Similarly, the total number of cycling cells (total Ki67^+^) increased at P7, but decreased significantly at P60; such a decrease occurred in dividing type-1 (Ki67^+^/GFAP^+^/nestin^+^), type-2ab (Ki67^+^/GFAP^−^/nestin^+^) and type-2b (Ki67^+^/nestin^+^/DCX^+^) progenitor cells, while type-3 (Ki67^+^/nestin^−^/DCX^+^) did not differ. Cell numbers in the dentate gyrus are mean ± SEM of the analysis of three animals per group. **(C)** Percentage of cells exiting the cell cycle (ratio between BrdU^+^/Ki67^−^ and total BrdU^+^ progenitor cells; *n* = 3 mice) after a BrdU pulse of 2 hours or of 20 h. *p < 0.05, **p < 0.01, or ***p < 0.001 vs. *Btg1*^+/+^ dentate gyrus; Student’s *t* test.

First, in the dentate gyrus of P60 mice, we measured the number of proliferating progenitor cells entering in S phase, identified by incorporation of a short BrdU pulse of 2 h (Arguello et al., 2008), and observed a non-significant decrease in *Btg1*-null mice (Figure 4B). Moreover, no change was observed in the number of progenitor cells in G2/M-phase, identified by the mitotic marker antiphospho-histone H3, PH3 (99.9 ± 22.5 and 95.3 ± 24.9 average cells ± SEM in whole dentate gyrus of *Btg1*^+/+^ and *Btg1*^−/−^ mice, respectively; Kaitna et al., 2002). This normal rate of BrdU incorporation was an unexpected result, as *Btg1* is a known antiproliferative gene (Rouault et al., 1992) whose ablation would be expected to increase the number of dividing cells. Therefore, we verified whether the BrdU incorporation did not change also at an earlier post-natal age, P7, when the proliferation rate is higher, and found that BrdU incorporation increased highly (34%, *p* = 0.002; Figure 4B). We further analyzed the total number of cycling progenitor cells by means of the proliferation marker Ki67 (Scholzen and Gerdes, 2000) and observed that at P60 Ki67^+^ cells, quite surprisingly, decreased significantly by 15%, whereas at P7 they increased significantly by about 55% (*p* = 0.0005 and *p* = 0.0000, respectively; Figures 4A,B). By analyzing each population of dividing progenitor cells at P60, we found that type-1 stem (Ki67^+^/GFAP^+^/nestin^+^) and type-2ab progenitor cells (Ki67^+^/GFAP^−^/nestin^+^, identified by the presence of nestin and absence of GFAP expression), decreased significantly in *Btg1* knockout mice (28% decrease, *p* = 0.014, and 40% decrease, *p* = 0.007, respectively; Figures 4A,B). Consistently, type-2b progenitor cells at P60 decreased significantly by 27% in *Btg1*-null mice with respect to *Btg1* wild-type mice (Ki67^+^/nestin^+^/DCX^+^; *p* = 0.049, Figures 4A,B), while no difference was observed in the number of dividing type-3 progenitor cells (Ki67^+^/nestin^−^/DCX^+^; Figures 4A,B). As shown in Figure 4A, the dentate gyrus of *Btg1*-null mice at P60 presents not only a decrease of Ki67-positive cells, but also an evident reduction of the nestin-positive and GFAP-positive arborization. As a whole, these data indicate that ablation of *Btg1*, while at an early post-natal age (P7) induced an increase of cycling dentate gyrus progenitor cells, in adult mice at P60 resulted in a stable, strong decrease of their number, suggesting the occurrence of a reduction of the pool of progenitor cells.

We reasoned that the normal rate of progression through the S phase of BrdU^+^ progenitor cells, as well as the parallel strong decrease of cycling Ki67^+^ progenitor cells observed in *Btg1*-null mice at P60, could also reflect a higher frequency of exit from the cell cycle after the S phase. We analyzed this possibility and found that in P60 mice the percentage of progenitor cells that exited cell cycle within either 2 or 20 h after the S phase – calculated as the number of BrdU-positive/Ki67-negative progenitor cells, divided by total number of BrdU-positive cells (Siegenthaler and Miller, 2005) – increased about 6.7-fold and 2.4-fold, respectively, in the *Btg1*-null dentate gyrus (BrdU^+^Ki67^−^/BrdU^+^ total cells; 2 h after BrdU pulse, *p* = 0.003; 20 h after BrdU pulse, *p* = 0.0008; Figure 4C). In contrast, at P7 a lower percentage of *Btg1*-null dentate gyrus progenitor cells exited the cell cycle (Figure 4C). The highly increased exit from cell cycle at P60 could in part explain the decreased number observed in adult *Btg1*-null mice of dividing progenitor cells and, consequently, of new 1- to 5-day-old neurons, but did not clarify the fate of the cells that exited the cell cycle.

### Ablation of *Btg1* drives the dentate gyrus pool of adult progenitor cells into apoptosis

**Figure 5:**
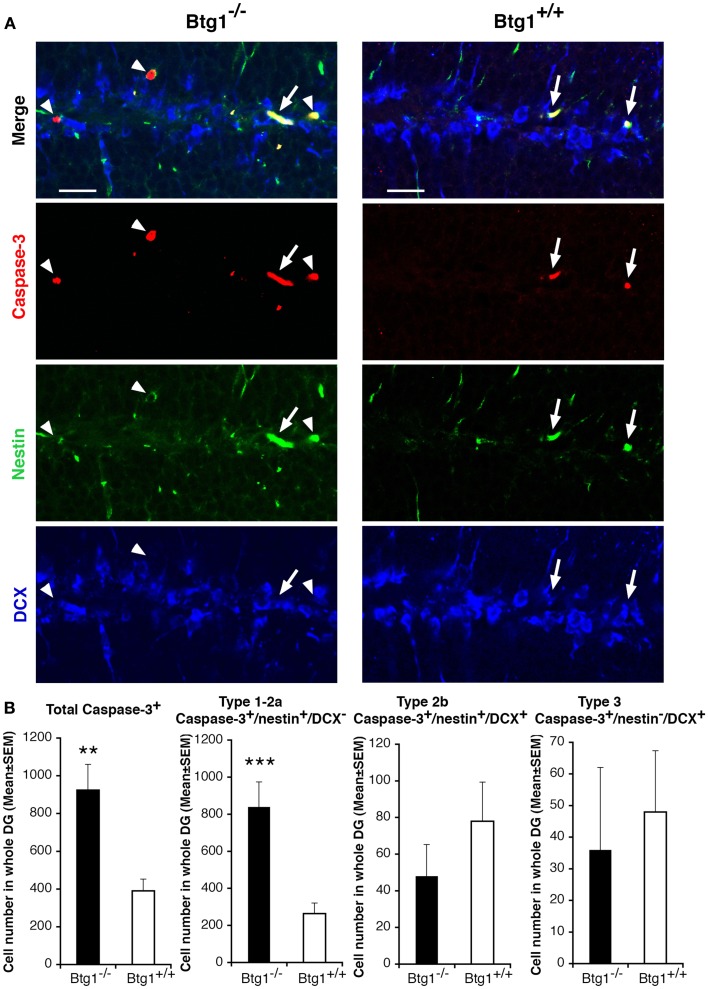
**Ablation of Btg1 induces a massive apoptosis in adult stem and progenitor cells of the dentate gyrus**. **(A)** Representative confocal images of apoptotic cells in the dentate gyrus of P60 *Btg1*^+/+^ and *Btg1*^−/−^ mice, showing either type-1 stem cells/type-2a progenitor cells (Caspase-3^+^/nestin^+^/DCX^−^; red, green and blue, respectively; white arrowheads) or type-2b progenitor cells (Caspase-3^+^/nestin^+^/DCX^+^; white arrows). Scale bar, 25 μm. **(B)** Analysis in the P60 dentate gyrus of the total number of apoptotic cells (Caspase^+^), and of type-1–2a (Caspase-3^+^/nestin^+^/DCX^−^), type-2b (Caspase-3^+^/nestin^+^/DCX^+^) and type-3 (Caspase-3^+^/nestin^−^/DCX^+^) stem and progenitor cell. Apoptosis was significantly higher in type-1–2a stem and progenitor cells. Cell numbers are mean ± SEM of the analysis of three animals per group. **p < 0.01, or ***p < 0.001 vs. *Btg1*^+/+^ dentate gyrus; Student’s *t*-test.

We thus analyzed the survival of dentate gyrus cells. We observed that in P60 *Btg1*-null mice the total number of apoptotic cells underwent a striking 2.4-fold increase with respect to those in wild-type, as detected by positivity to activated Caspase-3, marker of apoptosis (Nicholson et al., 1995; *p* = 0.001; Figures 5A,B). An analysis of the dentate gyrus progenitor populations showed that type-1 and type-2a progenitor cells of *Btg1*-null mice underwent apoptosis 3.2-fold more frequently (Caspase-3^+^/nestin^+^/DCX^−^; Figures 5A,B) than in wild-type mice, while a non-significant decrease appeared in type-2b (Caspase-3^+^/nestin^+^/DCX^+^; Figures 5A,B) and in type-3 progenitor cells (Caspase-3^+^/nestin^−^/DCX^+^).

### *Btg1*-null adult dentate gyrus progenitor cells exit the cell cycle expressing p53 and p21 and undergo apoptosis within 5 days after completing the S phase

**Figure 6:**
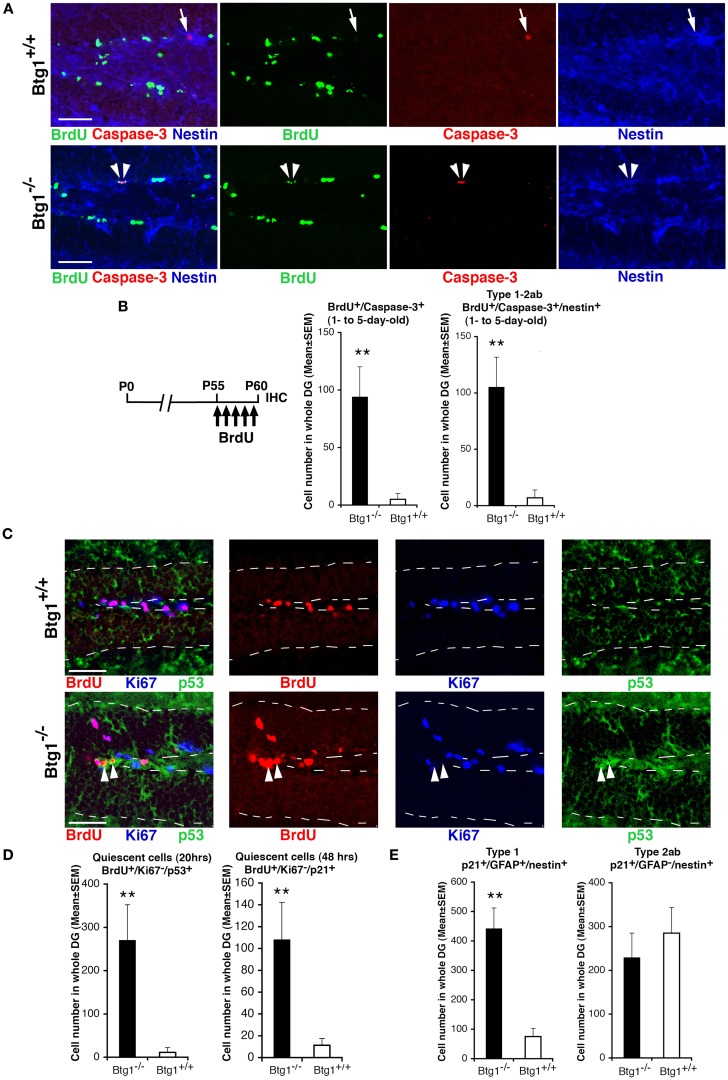
**Btg1-null adult stem and progenitor cells of the dentate gyrus undergo quiescence within 20 h and apoptosis within 5 days after entering the S phase**. **(A)** Representative confocal images showing apoptotic type-1–2ab progenitor cells triple-labeled BrdU^+^/Caspase-3^+^/nestin^+^ (indicated by white arrowheads) in the P60 *Btg1*-null dentate gyrus after a 5-day BrdU pulse. In *Btg1* wild-type dentate gyrus only BrdU^−^/Caspase-3^+^/nestin^+^ progenitor cells are detectable (indicated by a white arrow). Scale bars, 50 μm. **(B)** Corresponding scheme of BrdU treatment and quantification of cell numbers, showing an increase in the *Btg1*-null dentate gyrus of cells undergoing apoptosis within 5 days after entering S phase, either in the total progenitor cells population (BrdU^+^/caspase-3^+^) or in type-1–2ab cells (BrdU^+^/caspase-3^+^/nestin^+^). **(C)** Representative confocal images showing *Btg1*-null dentate gyrus cells that soon after entering the S phase (as monitored by a 20-h BrdU pulse) become quiescent, i.e., cells that have exited the cell cycle (Ki67^−^) and are p53-positive (BrdU^+^/Ki67^−^/p53^+^; red, blue, and green, respectively, indicated by white arrowheads). No BrdU^+^/Ki67^−^/p53^+^ cells are detectable in the *Btg1* wild-type dentate gyrus. Dotted lines show the boundaries of the dentate gyrus. Scale bars, 50 μm. **(D)** Increase in *Btg1*-null mice of progenitor cells becoming quiescent within 20 or 48 h after entrance into S phase, either p53-positive (BrdU^+^/Ki67^−^/p53^+^) or p21-positive (BrdU^+^/Ki67^−^/p21^+^), respectively. P60 mice were analyzed 20 or 48 h after a single BrdU injection, as indicated. **(E)** The expression of p21 increases in type-1 progenitor cells (p21^+^/GFAP^+^/nestin^+^) of *Btg1*-null mice; no change occurs in type-2ab progenitor cells (p21^+^/GFAP^−^/nestin^+^). **(C–E)** Cell numbers are mean ± SEM of the analysis of three animals per group. **p < 0.01 vs. *Btg1*^+/+^ dentate gyrus; Student’s *t*-test.

We further asked whether the striking increase of apoptosis observed for type-1 and type-2a progenitor cells in *Btg1*-null mice was correlated to the cell cycle progression. Hence, after a 5-day BrdU pulse in P60 mice, we analyzed 1- to 5-day-old BrdU^+^/Caspase-3^+^ and BrdU^+^/Caspase-3^+^/nestin^+^ (type-1 and type-2ab) cells, and observed a very large increase (15-fold) of them in *Btg1*-null mice (*p* = 0.005 and *p* = 0.001, respectively; Figures 6A,B). No BrdU^+^/Caspase-3^+^ cells were detected with BrdU pulses of shorter duration (data not shown). This demonstrates that *Btg1*-null type-1 and type-2ab progenitor cells undergo apoptosis within 1–5 days after completing the S phase, and – together with the observed increase of progenitor cells exiting the cell cycle – it raises the question as to whether apoptosis was caused by a defect in cell cycle control consequent to *Btg1* deletion. Thus, we analyzed the expression of *p53* and *p21*. *p53* is a key regulator of the cell cycle that inhibits cell cycle progression when a cellular stress occurs, such as a misregulation of the cell cycle, acting either directly or through its effector *p21*, a cyclin-dependent kinase inhibitor that arrests proliferation and leads the cell into a condition of senescence (Brady and Attardi, 2010; Qian and Chen, 2010; Erol, 2011). We observed in the dentate gyrus of P60 *Btg1*-null mice a major increase of BrdU^+^/Ki67^−^/p53^+^ and BrdU^+^/Ki67^−^/p21^+^ progenitor cells (analyzed after a 20- or 48-h BrdU pulse, respectively; *p* = 0.001 for p53^+^ and *p* = 0.004, for p21^+^ cells; Figures 6C,D), i.e., of progenitor cells that after the entrance in S phase (BrdU-positive) have then become quiescent or senescent, as indicated by the exit from the cell cycle (being Ki67-negative) concomitant with the expression of p53 or p21. No BrdU^+^/Ki67^−^/p21^+^ progenitor cells were detected after a BrdU pulse shorter than 48 h (data not shown), consistently with the notion that p21 upregulation is effected by p53. We further sought to check which type of progenitor cells expressed p21, and found in *Btg1*-null mice a significant increase of p21^+^ type-1 (nestin^+^/GFAP^+^/p21^+^; *p* = 0.002; Figure 6E) but not of type-2ab progenitor cells (nestin^+^/GFAP^−^/p21^+^; *p* = 0.53; Figure 6E). We also verified whether the increase of BrdU^+^/Ki67^−^/p21^+^ corresponded to an increase of senescent cells in *Btg1*-null dentate gyrus, however no difference was found by visualizing β-galactosidase that specifically marks senescent cells (Dimri et al., 1995; data not shown). This indicates that the p21^+^ (and p53^+^) progenitor cells entered quiescence without attaining a stable exit from the cell cycle (i.e., a senescent state). As a whole, this indicates that in P60 *Btg1*-null mice: (i) progenitor cells within 2–20 h after the entrance in S phase undergo a process of transient quiescence (see also Figure 4C), followed by apoptosis; (ii) the ablation of *Btg1* impairs neurogenesis in the dentate gyrus probably as a result of the massive apoptosis occurring in type-1/type-2a progenitor cells, which reduces the pool of quiescent and dividing progenitor cells downstream.

### In the SVZ ablation of *Btg1* reduces the generation of new adult neurons and increases the number of adult progenitor cells undergoing apoptosis

**Figure 7:**
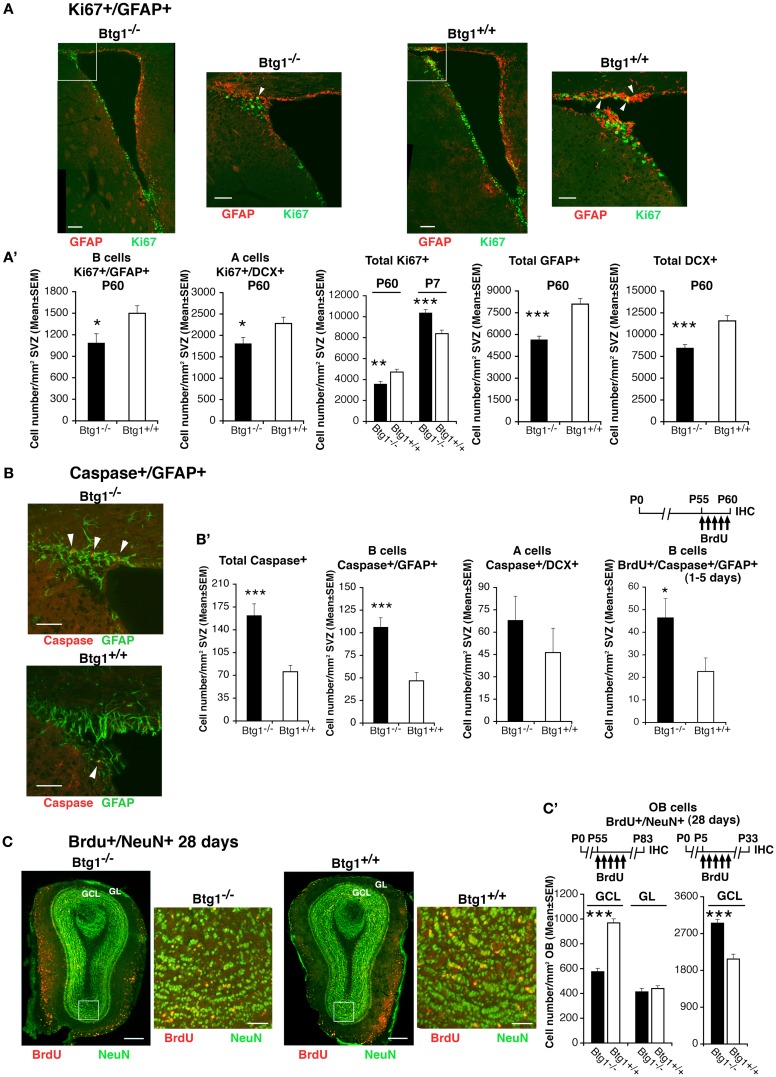
**Higher apoptosis frequency and decreased number of cycling stem/progenitor cells of the adult SVZ and of 28-day-old neurons of the adult olfactory bulb**. **(A)** Representative confocal images of coronal sections showing dividing B stem cells in the SVZ of P60 *Btg1*^+/+^ and *Btg1*^−/−^ mice, identified as double-labeled Ki67^+^/GFAP^+^ cells (green and red, respectively) and indicated by white arrowheads in the white box area at higher magnification. Scale bars, 100 and 50 μm (enlargement). **(A’)** Analysis in P60 mice (or in P7 mice where indicated) of the number per SVZ area of dividing B stem cells (Ki67^+^/GFAP^+^), of A neuroblast cells (Ki67^+^/DCX^+^) and total dividing cells (Ki67^+^), as well as of total B (GFAP^+^) and A (DCX^+^) cells. At P60 B and A cells decrease strongly, relative to wild-type. **(B)** Representative confocal images from P60 mice of apoptotic B stem cells (Caspase-3^+^/GFAP^+^; red and green, respectively; indicated by white arrowheads). Scale bars, 50 μm. **(B’)** Quantification of the number per area in P60 mice of apoptotic total cells and B stem cells, which decrease strongly in *Btg1*-null mice, and of A neuroblast cells. On the right: greater accumulation in the SVZ of *Btg1*-null mice of apoptotic 1- to 5-day-old B cells that have progressed into S phase (BrdU^+^/Caspase-3^+^/GFAP^+^). **(C)** Representative images (coronal sections) of 28-day-old terminally differentiated neurons in the adult olfactory bulb, after migration from the SVZ, identified as BrdU^+^/NeuN^+^ cells (red and green, respectively). BrdU^+^/NeuN^+^ neurons are detectable in the granule cells layer (GCL) and, in lower number, in the glomerular layer (GL); the higher magnification of the ventral GCL (white box) shows a lower number in *Btg1*-null mice of newly generated 28-day-old neurons, as indicated also [**(C’)**, graph on the left] by their quantification per area throughout the whole GCL (shown above is the scheme of treatment with five daily injection of BrdU performed from P55, 28 days before the analysis of the olfactory bulb). BrdU^+^/NeuN^+^ neurons in the GL did not significantly differ in number, relative to wild-type. [**(C’)**, graph on the right] On the contrary, 28-day-old SVZ neurons, analyzed in the GCL of olfactory bulb at an earlier postnatal age (see scheme above), are present in greater number in *Btg1*-null mice. Scale bars, 300 and 50 μm (enlargement). **(A’–C’)** Cell numbers are mean ± SEM of the analysis of three animals per group. *p < 0.05, **p < 0.01, or ***p < 0.001 vs. *Btg1*^+/+^; Student’s *t*-test.

We were further interested in evaluating whether the ablation of *Btg1* affected the generation of the neurons also in the SVZ, the other adult neurogenic niche (Alvarez-Buylla and Lim, 2004). We analyzed the number of dividing stem cells and neuroblasts by the proliferation marker Ki67, and observed that in P60 *Btg1*-null mice cycling type B astrocytic-like stem cells and type A neuroblasts, identified respectively by GFAP and DCX (Zhao et al., 2008), decreased significantly (about 28% decrease, *p* **=** 0.03, for B-cells; 21% decrease, *p* = 0.02, for A cells; Figures 7A,A’). Consistently, the total numbers of dividing cells (Ki67^+^) as well as of type B progenitor cells (GFAP^+^) and of type A neuroblasts (DCX^+^) decreased significantly (*p* = 0.006 for Ki67^+^ cells; Figures 7A,A’). Thus, the whole population of type B and A cells appeared strongly reduced in Btg1-null P60 mice. Conversely, the total number of cycling cells (Ki67^+^) in the SVZ of P7 *Btg1*-null mice increased significantly (*p* = 0.0001; Figures 7A,A’). In parallel, in SVZ of P60 *Btg1*-null mice the total number of apoptotic cells increased (2.1-fold; total Caspase-3^+^, *p* = 0.0002; Figures 7B,B’), more specifically type B apoptotic cells, within 5 days after birth (2.2-fold; Caspase-3^+^/GFAP^+^, *p* = 0.0001; BrdU^+^/Caspase-3^+^/GFAP^+^, *p* = 0.03 Figures 7B,B’). Moreover, the 28-day-old SVZ neurons at their final migratory destination in olfactory bulb, birth-dated by BrdU with five daily injection from age P55 and identified by the terminal differentiation marker NeuN, were highly reduced in adult *Btg1*-null mice, by about 40% in the granule cell layer (GCL, *p* = 0.000; Figures 7C,C’, see graph on the left and the scheme of BrdU treatment); in the glomerular layer (GL) no significant difference of BrdU^+^/NeuN^+^ neurons relative to wild-type was observed (*p* = 0.49; Figures 7C,C’**)**. On the contrary, the 28-day-old SVZ neurons that migrated in the olfactory bulb at an early post-natal age (identified by BrdU labeling from P5), increased significantly in *Btg1*-null mice (GCL, *p* < 0.0001; Figure 7C’, graph on the right and BrdU treatment scheme above). No significant difference of 28-day-old SVZ neurons labeled from P5 was observed in the GL, relative to controls (data not shown).

As a whole, this suggests that ablation of *Btg1* causes a transient increase in the generation of new neurons at an early post-natal age (P7), and then impairs neurogenesis in the adult SVZ, similarly to what we observed in the dentate gyrus, probably as a consequence of the massive apoptosis which the SVZ stem B astrocytic-like cells undergo.

### Loss of *Btg1* impairs proliferation and survival of SVZ neural stem cells

**Figure 8:**
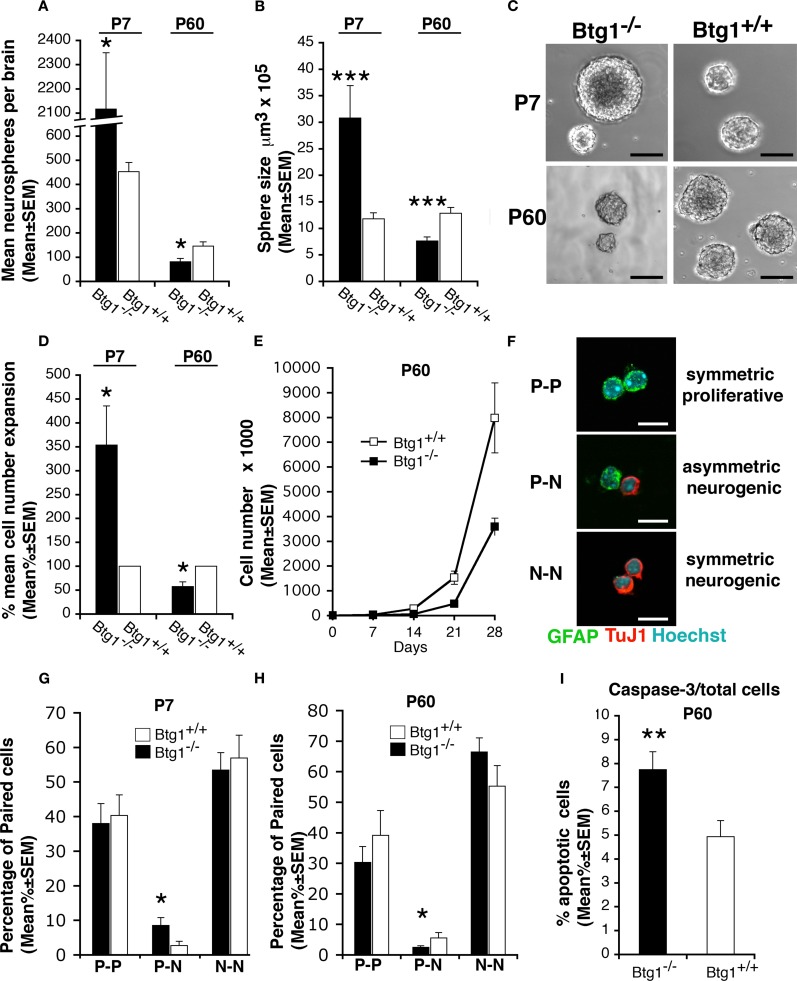
**Btg1 loss results in an initial expansion of neural stem cells in vitro, followed by an age-dependent decrease of proliferative capacity, self-renewal, and survival**. **(A)** Number (mean ± SEM) of clonal neurospheres derived from the subependyma of the lateral ventricle from *Btg1*-null and wild-type P7 or P60 mice (*n* = 4 and 5, respectively). Relative to control mice, neurospheres generated from P7 *Btg1*-null mice increased strikingly in number, while those generated from adult P60 mice decreased. **(B)** Volumes (mean ± SEM) of secondary neurospheres derived from *Btg1*-null and wild-type mice aged P7 or P60 (*n* = 4 and 5, respectively). With respect to wild-type mice, the volume of neurospheres from P60 *Btg1*-null mice was lower, after an initial increase observed in neurospheres from P7 mice. **(C)** Representative images of secondary neurospheres derived from *Btg1*-null and wild-type mice aged P7 or P60. Scale bars, 115 μm. **(D)** Percentage of cell expansion of primary neurosphere cultures from *Btg1*-null and wild-type mice (total number of cells at the end of culture divided by the initial number of cells; represented as mean percentage ± SEM, wild-type set to 100%). Relative to control, a greater expansion occurred in cells from P7 *Btg1*-null mice, whereas the expansion of cells derived from P60 *Btg1*-null mice was considerably lower (*n* = 4 and 5, respectively). **(E)** Growth curve displaying the amplification of 8000 cells derived from secondary neurospheres plated at *t*_0_, from P60 mice either *Btg1*-null or wild-type (*n* = 3). The amplification of *Btg1*-null cells is reduced in the long term, relative to wild-type cells. **(F)** Representative images of three different types of daughter-cells originating from individual NSPs from SVZ: two glial astrocytic-like proliferating progenitor cells (P–P; both labeled by GFAP), one glial astrocytic-like proliferating progenitor cell and one postmitotic neuron (P–N; labeled by GFAP and the neuronal marker TuJ1, respectively), and two postmitotic neurons (N–N; TuJ1^+^/TuJ1^+^). Scale bar 10 μm. **(G,H)** Quantification of the percentage of P–P, P–N, and N–N daughter-cells pair from SVZ of P7 and P60 *Btg1*-null and control mice. Cells counted: *n* = 198 and 330 for P7 *Btg1*^+/+^ and *Btg1*^−/−^ mice, respectively; *n* = 248 and 192 for P60 *Btg1*^+/+^ and *Btg1*^−/−^ mice (at least three mice per age). **(I)** Percentage of apoptotic cells in secondary neurospheres (mean percent ± SEM), detected as positive to activated Caspase-3. Cells from P60 *Btg1*-null presented a frequency 1.6-fold higher than control. *p < 0.05, **p < 0.01, or ***p < 0.001 vs. *Btg1*^+/+^; Student’s *t*-test.

To examine if the effect of the *Btg1* loss on NSPs proliferation and survival observed *in vivo* in this report is an intrinsic property of the cells, we performed proliferation studies on primary NSPs isolated from SVZ and grown in culture as neurospheres. NSPs, derived either from 7-day-old or 2-month-old *Btg1* wild-type and knockout mice, were plated at low density in order to perform a clonal neurosphere assay as a measure of the percentage of NSPs in the brain (Kippin et al., 2005). By counting the total number of neurospheres formed after 7 days in culture, we observed a significant increase in the neurosphere-forming cell population derived from *Btg1*-null P7 mice, and a decrease in neurosphere-forming cell population from P60 mice, relative to wild-type (*p* = 0.01 at P7, *p* = 0.04 at P60; Figure 8A). This result could either reflect or recapitulate *in vitro* what we had already observed *in vivo*, i.e., an age-dependent reduction in the number of NSP cells in *Btg1* knockout vs. wild-type mice, and/or suggest that knockout cells from P60 mice proliferate less than their wild-type counterpart. To better investigate this aspect, we analyzed the frequency of symmetric and asymmetric cell division occurring within NSP in both mice, by performing secondary neurosphere assays (Reynolds and Weiss, 1996; Reynolds and Rietze, 2005). Under normal circumstances, asymmetric division maintains the population of NSPs at the same size; alternatively, symmetric division occurs when NSP generate two NSPs progeny, thereby expanding the population of NSPs. We therefore dissociated primary neurospheres, seeded them at low density, and measured the size (indicative of asymmetric division) of secondary spheres formed after 7 days *in vitro*. We found that in the absence of *Btg1* secondary neurospheres from P7 *Btg1*-null mice were larger, while neurospheres from adult (P60) *Btg1*-null mice were smaller (reflecting fewer asymmetric divisions), compared to their wild-type counterpart (*p* = 0.000 at P7 and P60; Figures 8B,C). In addition, we observed that the amplification capacity of *Btg1*-null neurospheres increased in neurospheres from P7 mice, whereas it decreased in neurospheres from P60 mice (*p* = 0.02 at P7, *p* = 0.04 at P60; Figure 8D). Similarly, a decrease was observed in the long term expansion of neurospheres from P60 *Btg1*-null mice, measured by growth curves (Figure 8E). These data suggest that the loss of *Btg1* affects both the self-renewal and the proliferative capacity of NSPs, causing a severe depletion of the stem cell compartment in adult mice.

To further analyze the influence of *Btg1* ablation on the mode of division of NSPs, we used the clonal pair cell assay that allows one to distinguish the relative changes in symmetric vs. asymmetric division of primary NSPs isolated from SVZ (Bultje et al., 2009). We observed that the fraction of NSPs that divided asymmetrically (giving one proliferating GFAP^+^ cell and one differentiated TuJ1^+^ neuron) significantly increased (threefold) in cultures from *Btg1*-null P7 mice relative to wild-type, whereas it significantly decreased (60%) in cultures from *Btg1*-null P60 mice (*p* = 0.04 at P7 and at P60; Figures 8F–H). No significant difference was observed in symmetric divisions in primary NSPs from P7 or P60 mice. This indicated an age-dependent decrease of asymmetric divisions.

Since stem cells exhaustion could also depend on apoptosis, we also wanted to measure apoptotic cell death in *Btg1*-null neurospheres by looking at the expression of the apoptotic-specific marker activated caspase-3. We found significantly more active caspase-3-positive cells in *Btg1*-null neurospheres from 2-month-old mice compared to the wild-type ones (56% increase, *p* = 0.005; Figure 8I). Taken together, our results demonstrate that *Btg1* is required for the proper self-renewal of the neural stem cells, since in the absence of *Btg1* we observed a decrease in cell proliferation and an increase in apoptotic cell death.

### Defective hippocampus-dependent learning in *Btg1*-null mice

**Figure 9:**
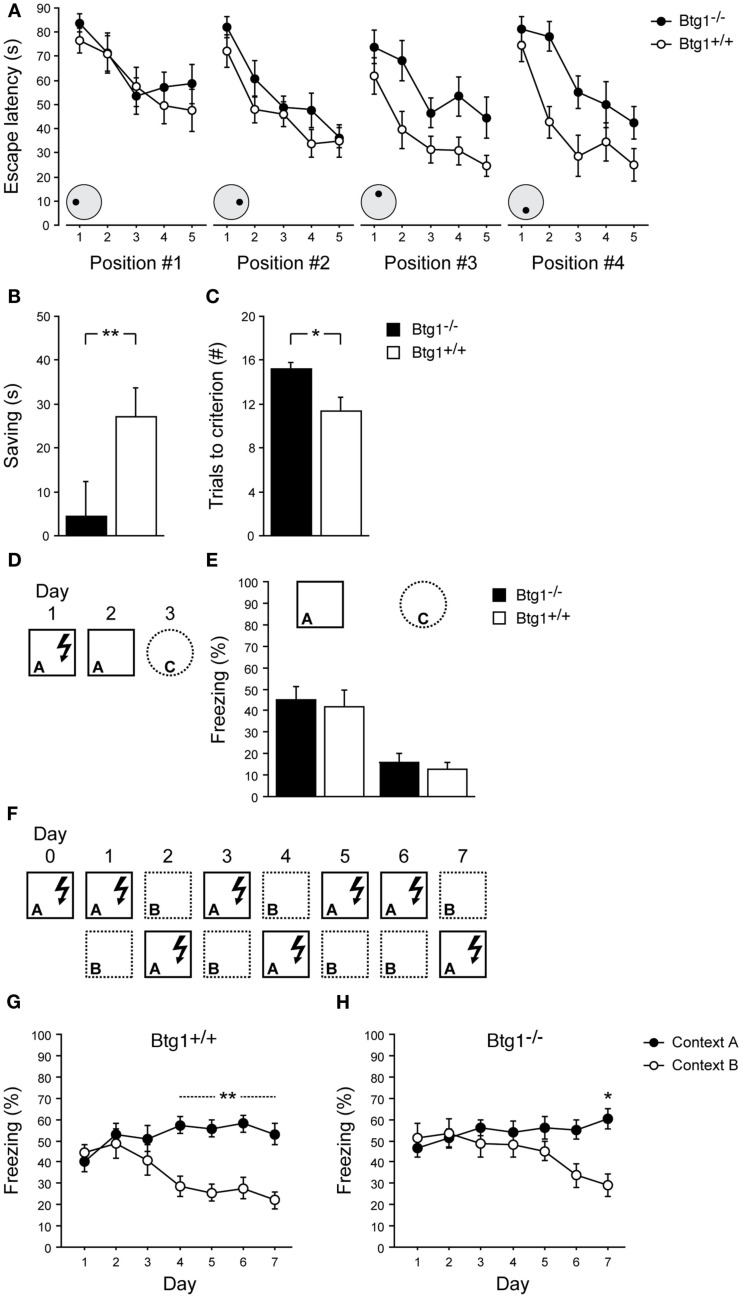
**Learning abilities of Btg1-null mice**. *Water maze*. **(A)** Escape latencies of the first five trials for each of the four successive platform locations. **(B)** The reduction of escape latencies (saving) animals achieved as they passed from the first to the second trial of the third and fourth training sessions (averaged). **(C)** The number of trials animals needed to reach the performance criterion in the third and fourth training sessions (averaged). *Contextual fear conditioning*. **(D)** Experimental procedure to test one-trial contextual fear conditioning. **(E)** Upon re-exposure to the shock-associated context, both *Btg1*-null and wild-type mice showed equally increased levels of freezing behavior; conversely, negligible freezing was detected in a different context. **(F)** Experimental procedure to test contextual fear-discrimination learning. **(G)** Wild-type mice were able to discriminate between the shock-associated context and the similar context by day 4 of testing, which was stably maintained until day 7; **(H)** Conversely, *Btg1*-null mice started discriminating by day 6, with difference in freezing behavior reaching statistical significance only by day 7. Results are presented as mean ± SEM. *p < 0.05; **p < 0.01.

Learning and memory of *Btg1*-null mice were firstly assessed by a delayed matching-to-place water maze protocol, which has been used by Chen et al. (2000) and Zeng et al. (2001) to assess rodents’ ability to perform one-trial learning and episodic-like memory. As training progressed, both *Btg1*-null (*n* = 12) and control (wild-type; *n* = 12) mice retained their ability to locate the hidden platform at each of the four positions it was sequentially moved to [Figure 9A, left to right; effect of trial, for all positions: *F*(4, 88) > 5.75, *p* < 0.001; trial × genotype interaction, for all position: *F*(4, 88) < 1.10, *p* > 0.361; two-way repeated measures ANOVA]. However, while no effect of genotype was observed for the first and the second platform positions [Figure 9A, positions #1 and #2; effect of genotype, for both positions: *F*(1, 22) < 2.49, *p* > 0.129], a statistically significant difference between genotypes emerged for the third and fourth positions, with *Btg1*-null mice reducing their escape latencies at a slower rate compared to wild-type mice [Figure 9A, positions #3 and #4; effect of genotype, for both positions: *F*(1, 22) > 10.99, *p* < 0.003]. Consistently, a statistically significant difference between genotypes was observed in the reduction of escape latencies animals achieved as they passed from the first to the second trial of the last two training sessions (Figure 9B; *p* = 0.039; Student’s *t*-test), while maintaining comparable swimming speed (*p* = 0.617) and thigmotaxis (*p* = 0.566). Furthermore, *Btg1*-null mice needed a significantly higher number of trials to reach the performance criterion over the last two training sessions, compared to wild-type mice (Figure 9C; *p* = 0.003; Student’s *t*-test).

The ability of *Btg1*-null mice to differentiate between overlapping contextual representations was further assessed by a contextual fear-discrimination learning task (McHugh et al., 2007; Sahay et al., 2011a). *Btg1*-null (*n* = 8) and control (*n* = 8) mice were preliminarily submitted to a single-trial footshock-context pairing procedure (Figure 9D). Upon re-exposure to the conditioning chamber (A), 24 h after being trained, both groups showed equally increased levels of freezing behavior; conversely, negligible freezing was detected in a distinct context (C), 48 h after training [Figure 9E; effect of genotype: *F*(1, 14) = 0.50, *p* = 0.490; effect of context: *F*(1, 14) = 94.16, *p* < 0.001; context × genotype interaction: *F*(1, 14) = 0.01, *p* = 0.91; two-way repeated measures ANOVA]. Independent groups of mice were subsequently tested for contextual fear-discrimination learning by prolonged training in two similar contexts (A and B), with footshock delivered only in one (Figure 9F). Both *Btg1*-null (*n* = 10) and wild-type (*n* = 10) mice showed generalization between the two contexts during the early days of training, and were able to discriminate at the end; however, while wild-type mice gained effective discrimination on day 4, which was stably maintained until day 7 (*p* < 0.010), *Btg1*-null mice started discriminating by day 6 (*p* = 0.074), with difference in freezing behavior evoked by the two contexts reaching statistical significance only by day 7 (*p* = 0.013) [Figure 9G; training × context interaction: *F*(6, 108) = 4.90, *p* < 0.001; Figure 9H; training × context interaction: *F*(6, 108) = 3.62, *p* = 0.003; two-way repeated measures ANOVA followed by analysis of simple effects].

Overall, these data indicate that the basic ability to encode contextual features is preserved in Btg1 knockout mice, which makes them able to differentiate among markedly dissimilar contexts; by contrast, their finer mnemonic discrimination appears to be impaired, when challenged by subtle differences in contextual details to be promptly distinguished.

## Discussion

Understanding the molecular pathways controlling neural stem cells self-renewal and maintenance may shed light on tissue homeostasis in the neurogenic niches during adult neurogenesis. This study addresses the role of the antiproliferative gene *Btg1* in modulating neurogenesis in the adult brain. Our data clearly indicate that *Btg1* plays a specific role in regulating neural stem cells proliferation and subsequently their quiescent state as well as their survival. We report that the loss of *Btg1* causes an increased proliferation of newborn neurons in an early post-natal age, followed by a decline of neurogenesis in the adult, associated with death by apoptosis. In particular, we observe in the neurogenic niches (subgranular zone of the dentate gyrus and SVZ) of P7 knockout mice that the number of stem cells and progenitors positive for the two proliferation markers Ki67 and BrdU is significantly higher relative to control, whereas in the adult mice the rate of proliferation strongly decreases, especially in the dividing type-1 stem and type-2 progenitor cells of the dentate gyrus.

### Control of cell cycle in dentate gyrus stem cells by *Btg1*: Its ablation impacts on quiescence, survival, and proliferative capacity

These effects can be primarily originated by the loss of the antiproliferative action of *Btg1*, whose deprivation is sufficient to induce an initial expansion of neural stem cells, followed by reduced proliferative capacity and depletion of the population of stem cells, and by the decline of the adult neurogenesis with age.

Given that the cell cycle transition from G1 to S phase is enhanced at P7 in *Btg1*-null progenitor cells, while the G2/M transition appears not altered (as PH3 labeled cells do not change in *Btg1*-null mice at either age; data not shown), our findings suggest that stem and progenitor cells lacking *Btg1* are defective in the control of the progression from G1 to S phase. This possibility is consistent with the known ability of *Btg1* to arrest the cell cycle in G1 phase (Li et al., 2009). It is well known that the inactivation of molecules regulating the transition from G1 to S phase, for instance pRb, can raise a conflict between ongoing proliferative and proliferation-inhibitory stimuli, leading to cell death (Lee et al., 1994).

Thus, the massive apoptotis of the pool of stem type-1 cells and of transit amplifying progenitor type-2a cells, likely triggered by the absence of the negative control of cell cycle exerted by *Btg1*, appears to be one cause of the decrease of adult neurogenesis. Apoptosis can in fact account, at least in part, for the reduction observed in the number of dividing (Ki67-positive) type-1 and type-2ab progenitor cells and can plausibly contribute in the long period to deplete the pool in the adult mice. Stem type-1 cells are mainly quiescent and divide only slowly, as they account for only 5% of the total number of dividing cells in the dentate gyrus (Filippov et al., 2003; Kronenberg et al., 2003). Consistently, we show that in P60 mice type-1 stem and type-2ab progenitor cells (nestin-positive cells) undergo apoptosis 1–5 days after completing the S phase, as observed after a BrdU pulse of 5 days (BrdU^+^/caspase-3^+^/nestin^+^ cells). No apoptosis is detectable at earlier periods, such as after a 20-h pulse of BrdU (data not shown), indicating the occurrence of a progressive and slow accumulation of apoptotic progenitor cells. However, progenitor cells in *Btg1*-null adult dentate gyrus exit the cell cycle as early as 2 h after the completion of the S phase (BrdU^+^/Ki67^−^ cells) and within 20 h express p53 and within 48 h p21 (BrdU^+^/Ki67^−^/p53^+^ or BrdU^+^/Ki67^−^/p21^+^ cells). Thus, considering that the length of cell cycle in dentate gyrus progenitor cells is about 14 h (Mandyam et al., 2007), we hypothesize that dividing progenitor cells, chiefly type-1 stem and type-2a progenitor cells, enter a quiescent state soon after completing the S phase, followed, within a few days, by apoptosis.

Indeed, the observed increase of cell cycle exit, i.e., the entrance into quiescence, and the increase of apoptosis occurring in progenitor cells within a few days after birth, can be accounted for by the induction in *Btg1*-null newborn cells of the key negative regulator of cell cycle *p53* and its effector *p21*. In fact, any cellular stress signal, such as a misregulation of cell cycle, activates *p53* in a specific manner by post-translational modifications (Qian and Chen, 2010). *p53* activation leads to either cell cycle arrest and senescence – through *p21*, its major effector of growth arrest and senescence – or to apoptosis (Kruse and Gu, 2009; Qian and Chen, 2010). It has been shown that mice lacking *p53* display an elevated proliferation rate in the adult neurogenic niches associated with an increase in self-renewal and apoptosis (Meletis et al., 2006), while an overexpression of *p53* affects the proliferation of stem and progenitor cells in adult neurogenesis (Medrano et al., 2009). *p21* plays a pivotal role in the maintenance of quiescence in adult neural stem cells, a mechanism ensuring a fine regulation of the turnover of the stem cell niche throughout the lifespan (Kippin et al., 2005). This is consistent with our data indicating that *Btg1*-null progenitor cells attain a quiescent, likely transient state of exit from cell cycle.

Notably, not less critical than apoptosis, an age-dependent intrinsic decrease of the replicative potential of stem and progenitor cells may be involved in the observed decrease of the number of proliferating progenitor cells. In fact, the absolute number of proliferating *Btg1*-null progenitor cells that undergo apoptosis in the dentate gyrus at P60 is only a fraction of the cells that have ceased proliferating (total BrdU^+^); furthermore, the percentage of *Btg1*-null progenitor cells exiting the cycle is low at P7 but very high at P60, relative to controls. A decrease of the replicative potential has also been implicated in the knockout of the cyclin-dependent kinase inhibitor *p21* (Kippin et al., 2005).

In summary, our findings are compatible with a model where the lack of the negative control of cell cycle by *Btg1* triggers in the pool of nestin^+^ stem-like type-1 cells and in type-2a progenitor cells of the dentate gyrus an accelerated division rate with reduced exit from cell cycle, as observed at P7, and the counteracting activation of the cell cycle inhibitors *p53* and *p21*. Consequently, in the long period (at P60) dividing type-1 cells – i.e., the self-renewal pool – and transit amplifying type-2ab progenitor cells become less numerous and partially depleted, possibly because of the concomitant progressive reduction of replicative potential and increased apoptosis. Altogether, such a decrease of the self-renewal pool results in a strong reduction of 1- to 5-day-old type-3 (BrdU^+^/nestin^−^/DCX^+^) progenitor cells and of the whole population of mature neurons stage 5 and 6 (DCX^+^ and DCX^+^/NeuN^+^).

### *Btg1* ablation reduces the self-renewal and proliferative capacity also of SVZ stem cells

A very similar situation is observed also in the SVZ of adult *Btg1*-null mice, where the dividing type B GFAP^+^ stem-like cells (Ki67^+^/GFAP^+^ cells) decrease in number and undergo apoptosis; a lower number of terminally differentiated 28-day-old neurons is also detected in their final migratory destination, the olfactory bulb. As observed in the dentate gyrus, this decrease of neurogenesis in adult SVZ is preceded in the early post-natal SVZ of *Btg1*-null mice by a transient increase of proliferating cells and of terminally differentiated neurons (28-day-old) detected in the olfactory bulb.

Moreover, neurospheres generated from neural stem and progenitor cells isolated from SVZ of *Btg1*-null mice show an age-dependent decrease of proliferative capacity, with a reduction of the ability to replicate by asymmetric division, responsible for self-renewal. Thus, the emerging profile of *Btg1* appears to be that of a gene responsible for the control of the self-renewal and quiescence of the pool of stem and progenitor cells, acting primarily by regulating cell cycle progression and, in consequence, survival. This control by *Btg1* impacts on the generation and differentiation of dentate gyrus and SVZ progenitor cells and neurons. We cannot exclude that the observed decrease of the number of stage 5 and 6 dentate gyrus post-mitotic neurons might in part depend on a requirement of *Btg1* not only for maintenance and survival of the pool of progenitor cells, but also for differentiation, as already observed in knockout mice of the family related gene *PC3*/*Tis21* (Farioli-Vecchioli et al., 2009). It appears, however, that unlike *Btg1*, the main requirement of *PC3*/*Tis21* in the adult hippocampus is for terminal differentiation of stage 6 neurons, where *PC3*/*Tis21* is expressed (Attardo et al., 2010), rather than for cell cycle progression. In fact, after ablation of *PC3*/*Tis21* the pool remains intact, given that only type-3 progenitor cells show a moderate enhancement of proliferation, probably connected to the control of the asymmetric division preceding terminal differentiation (Farioli-Vecchioli et al., 2009).

### Reduced neurogenesis following *Btg1* ablation impairs pattern separation

The loss of progenitor cells and terminally differentiated neurons in the dentate gyrus of *Btg1*-null mice was associated to impairment in hippocampus-dependent learning and memory, namely, a failure in delayed matching-to-place version of the Morris water maze, designed to assess episodic-like components of memory (Chen et al., 2000; Zeng et al., 2001), and a failure to rapidly distinguish between similar settings when trained in a contextual fear-discrimination learning task (McHugh et al., 2007).

This is consistent with the knowledge that adult neurogenesis could ultimately contribute to enhance the extent of information encoded by the dentate gyrus and improve pattern separation, which refers to the ability to discriminate among potentially overlapping experiences (Aimone et al., 2011; Sahay et al., 2011b), identified as intrinsic to episodic memory (Tulving, 2002; Yassa and Stark, 2011).

## Conflict of Interest Statement

The authors declare that the research was conducted in the absence of any commercial or financial relationships that could be construed as a potential conflict of interest.
